# Sprint Running Coordination: A Dynamical Systems Perspective

**DOI:** 10.1007/s40279-025-02380-6

**Published:** 2026-01-13

**Authors:** Dylan S. Hicks, Stuart McMillan, Wolfgang Schöllhorn, Roland van den Tillaar

**Affiliations:** 1 Flinders University Institute for Mental Health and Wellbeing, College of Education, Psychology and Social Work, Flinders University, Bedford Park, SA Australia; 2 Altis, Phoenix, AZ USA; 3 Johannes Gutenberg-University of Mainz, Institute for Sport Science, Mainz, Germany; 4 Department of Sport Science and Physical Education, Nord University, 93, Levanger, 7601 Norway

## Abstract

Sprinting is a complex skill required in many team and individual sports, with practitioners placing an emphasis on enhancing this aspect of performance to improve sporting success. The task of sprinting involves patterns of inter- and intra-limb coordination and control, which emerge as the athlete accelerates to their maximal velocity. Traditionally, practitioners have attempted to modify sprint coordination patterns from a reductionist or cognitive perspective, decomposing performance to individual component parts using knowledge of coaching literature, biomechanics and skill acquisition theory. However, this approach widely neglects the dynamic and complex interactions that shape sprinting more holistically. This perspective article presents sprint coordination within a dynamical systems theory framework, emphasising how sprint performance emerges from constantly varying internal and/or external boundary conditions that regulate patterns of coordination by controlling mechanical, metabolic and neurophysiological degrees of freedom within the limits of the system. Thereby, movement variability is viewed as an essential component of coordination rather than simply ‘noise’. We also review classification schemes that identify stable sprint coordination patterns or strategies, with an emphasis on the acceleration and maximal velocity phases. We then examine practices towards “optimal” sprint technique, plus consider coordinative processes including self-organisation, phase transition and shifts in attractor states, alongside skill acquisition approaches used to establish functional sprint coordination patterns. Ultimately, we aim to present an alternative view for sprint practitioners to consider the complexities of sprint coordination and performance through a dynamical systems lens.

## Key Points

**Table:** 

For sprint practitioners, shifting from reductionist thinking towards ‘systems’ thinking, encourages a holistic understanding of how sprint performance emerges from interaction within a complex adaptive system.
A dynamical systems perspective highlights the importance of coordinative structures, degrees of freedom and movement variability in understanding sprint coordination.
Overall, we challenge practitioners to explore skill acquisition approaches embedded in dynamical systems theory to develop stable functional movement patterns, enabling athletes to achieve individually, optimal sprint coordination and performance.

## Introduction

Sprint coaches continually strive to enhance performance by modifying coordination patterns to enhance sprint running technique [1]. Most sprint coaching literature describes the action as a ‘skill’, suggesting the rhythmical coordination pattern can be learned [2–5], often through structured drills and exercises. Typically, a major emphasis is placed on improving coordination patterns during the acceleration and maximal velocity phases of sprinting as they are key aspects informing sprint performance [6, 7]. Whereas, in many field and court-based sports such as football, basketball and rugby, sprint coordination patterns are reinforced through association with goal-scoring or attacking opportunities [8, 9]. Given the prioritisation of these coordination patterns in training programmes, it is critical to explore whether current approaches sufficiently explain the complex mechanical and neurophysiological processes underlying sprint performance.

Attempts to modify sprint coordination and performance have generally occurred through what may be described as reductionist biomechanical models [5, 10–12]. These approaches often identify averaged group-oriented sprint coordination strategies or patterns to improve efficiency of movement and achieve ‘optimal’ sprint technique [13, 14]. Recent sprint biomechanics research, including a systematic review consisting of 109 studies [7], quantified force orientation during specific sprints phases [15–19], force–velocity-power relationships [20] and intra-limb coordination patterns [21–24], as key sprint-specific variables that practitioners should target to refine sprint mechanics. Further investigations into sprint coaching theory and methodology [4, 5, 12, 25–29] have identified training methods and principles, plus instructional cues to enhance sprint technique and improve proprioception during the movement task [1]. Additionally, these approaches generally underpin sprint coaching pedagogy and psychology, where a traditional cognitive perspective to skill acquisition is followed, which places a focus on the enhancement of specific coordination patterns through rote repetition of sprint drills and corrections [30]. This competency-based skill progression assumes movement variability is to be minimised, and athletes are directed towards a singular optimal technique that enhances sprint performance [31]. Despite these descriptions, attempts to understand and influence coordination changes from this perspective fails to explain the dynamic nature of ‘how’ coordination emerges within biological systems.

Given these limitations, alternative explanations of sprint coordination are warranted. We propose that the dynamical systems theory (DST) offers an appropriate framework upon which to analyse sprint coordination, an approach previously explored in human locomotion research [32–34]. Using DST, the human body is modelled as a ‘system’ with specific boundary conditions,^1^ consisting of several interacting sub-components (e.g. biomechanical, neurophysiological, psychological, cultural), where dynamic coordination occurs via a process of self-organisation, for example, a spatial, temporal or functional structural change to the system without specific information about the solution from outside of the system [37, 38]. Compared to the linear reductionistic approach, the sub-components’ non-linear interactions and changes over time are considered within a DST perspective. Furthermore, DST presents a framework for movement control and motor learning when working with complex systems [34], plus offers an inter-disciplinary approach to how sprint coordination emerges in the human motor system [31, 39–41].

Descriptions of coordination that appear in the sprint coaching literature are often linked to a person- and time-independent optimal technique and the step-by-step technical execution of specific sprint movements [1, 42]. However, these descriptions contrast the terminology and definitions used in scientific literature, where coordination is explored through a wider lens incorporating principles from motor learning [43]. Newell [44] defines coordination as relative motions between the same or opposing limb, whereas control is defined as the magnitude of limb movement, relative to the motion. Bernstein [45] highlighted coordinated movements during complex tasks occur due to various mechanical degrees of freedom, for example, joints, muscles and connective tissue, combining to achieve a functional task at each spatiotemporal scale. A further step towards explaining coordination is suggested by combining the mechanical degrees of freedom to the formation of coordinative structures [43, 46], thereby allowing the organism to achieve the same goal using different degrees of freedom (e.g. motor equivalence) or use the same degrees of freedom to complete different movement goals [32, 36]. This complex mechanical interplay is therefore critical to understanding the coordinative processes during a movement action such as sprinting.

Despite a DST approach becoming more prevalent within sport research [31, 47–50], translating this knowledge to the practitioner ‘on the ground’ remains a challenge. Therefore, this perspective paper aims to provide a synopsis for sprint practitioners to consider the complex processes of coordination within the skill of sprinting. We begin by discussing the theoretical background of systems approach in Sects. 2 and 3. Within these sections, we introduce DST as a framework to view sprint coordination changes within the systems boundary conditions and conclude by exploring the role constraints^2^ play in shaping sprint performance. In Sects. 4 and 5, we explore how coordination is quantified and classified in acceleration and maximal velocity sprinting and analyse how coordination is presented in the scientific and coaching literature. In Sects. 6 and 7, we critically reflect on the concept of optimal technique and relate concepts of self-organisation, stability and movement variability, as well as their role in sprint coordination patterns. Finally, we discuss skill acquisition concepts in sprinting in Sect. 8 and provide actionable insights and practical implications in Sect. 9. Overall, this perspective paper serves to bridge the gap between theory and practice, providing a comprehensive understanding of sprint running coordination.

## Theoretical Foundation: A Systems Approach

Research in sports medicine and performance has increasingly used a ‘systems approach’ to analyse the characteristics and behaviour of athletes, who are conceptualised as ‘complex systems’ [32, 48–50, 53]. Systems theory, first described by biologist Ludwig Von Bertalanffy as ‘general systems theory’ [54, 55] provided a framework to analyse a system as a whole consisting of various sub-components, with interacting constraints and distinct boundary conditions, which make it unique from all other systems. Rather than viewing system components in isolation, a systems framework explores the relationships and interactions between sub-components [56]. Since its introduction, there has been an increasingly differentiated characterisation of system classes, which refer either to their interaction with their surrounding environment (e.g. self-sufficient, closed, open), to the number, type and relationships of their sub-elements (e.g. simple, complicated, chaotic, complex) or to their temporal behaviour (e.g. static, dynamic, adaptive, predictable, unpredictable) as well as various combinations of these.

**Fig. 1:**
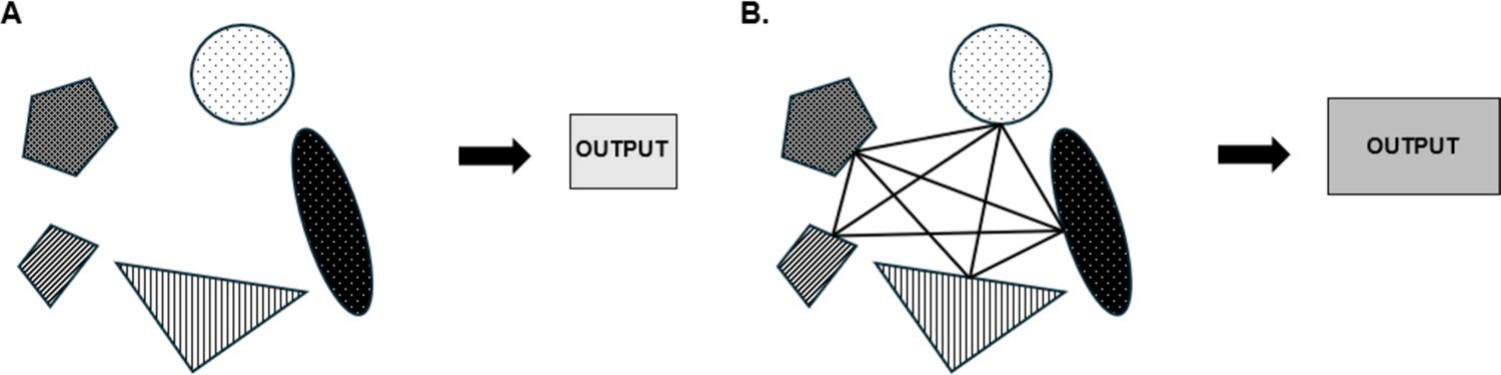
**A** Reductionist perspective of system sub-components working in isolation contributing to a smaller performance output.** B** A complex adaptive system with interacting sub-components, where when analysed over time, the system as a whole, for example, performance output, is greater than the individual sum of the sub-component parts

Correspondingly, an athlete is viewed as a complex, adaptive and unpredictable system [57], where coordination dynamics suggests an approach to describe how patterns of coordination can emerge over time [58]. Within the context of sprinting, relevant sub-components of the system include the biomechanical, physiological, technical, tactical and psychological processes. These sub-components are interdependent and their collective behaviour result in coordinated spatio-temporal patterns during sprinting [59]. Importantly, we suggest sprint performance does not arise from the isolated or linearly summed-up contribution of these sub-components, but from their dynamic interactions [60]. Reductionist perspectives most often consider each sub-component in isolation, whereas a complex systems perspective models performance that emerges from interactions of the organism and environment, producing a behavioural output that exceeds the sum of its individual parts, illustrated in Fig. 1.

A defining property of complex systems is their non-linearity, highlighting how little causes can contribute to large effects and vice versa [31, 34, 61]. This principle, metaphorically described by the ‘butterfly effect’, demonstrates how small fluctuations in one sub-system may cascade into large-scale outcomes in another, for example, a simple flap wing can cause a thunderstorm [62]. Such non-linear cause and effect relationships have previously been observed during running and sprinting. For instance, at the onset of fatigue (physiological), subtle changes in velocity (biomechanical) often lead to spontaneous shifts in coordination patterns (technical) and muscle activation synergies in an attempt to maintain performance [13, 63, 64]. These interactions demonstrate how coordination patterns dynamically adapt throughout running and sprint efforts in response to the application of force and step kinematics [65–68], reflecting the continuous interactions between multiple interconnected systems and their sub-components within them.

Dynamical systems theory advances complex systems understanding, by analysing spatio-temporal characteristics of coordination patterns in tasks requiring the use of multiple biomechanical degrees of freedom [69]. Within the broader principles of complex systems, DST explores how movement patterns emerge through a process of self-organisation and where movement variability is viewed as essential motor behaviour, rather than a movement error. Dynamical systems theory has been widely used to analyse coordination in complex motor skills [32, 48–50, 53, 70] and is particularly valuable for explaining learning processes [40, 71] in which new coordination patterns evolve in response to interacting boundary conditions, such as sequences of coach instructions that do not contain specific information for a possible solution. Dynamical systems theory also provides scope to quantitatively describe topological movement pattern changes at the individual level rather than averaging movement metrics across groups, offering insights beyond pre-post mean comparisons [72]. Importantly, DST emphasises the role boundary conditions play in shaping movement solutions that emerge during sprinting actions, thereby exploring shifts in coordination that emerge during sprint actions under varying boundary conditions of the system [31].

## Shaping Sprint Coordination

**Fig. 2:**
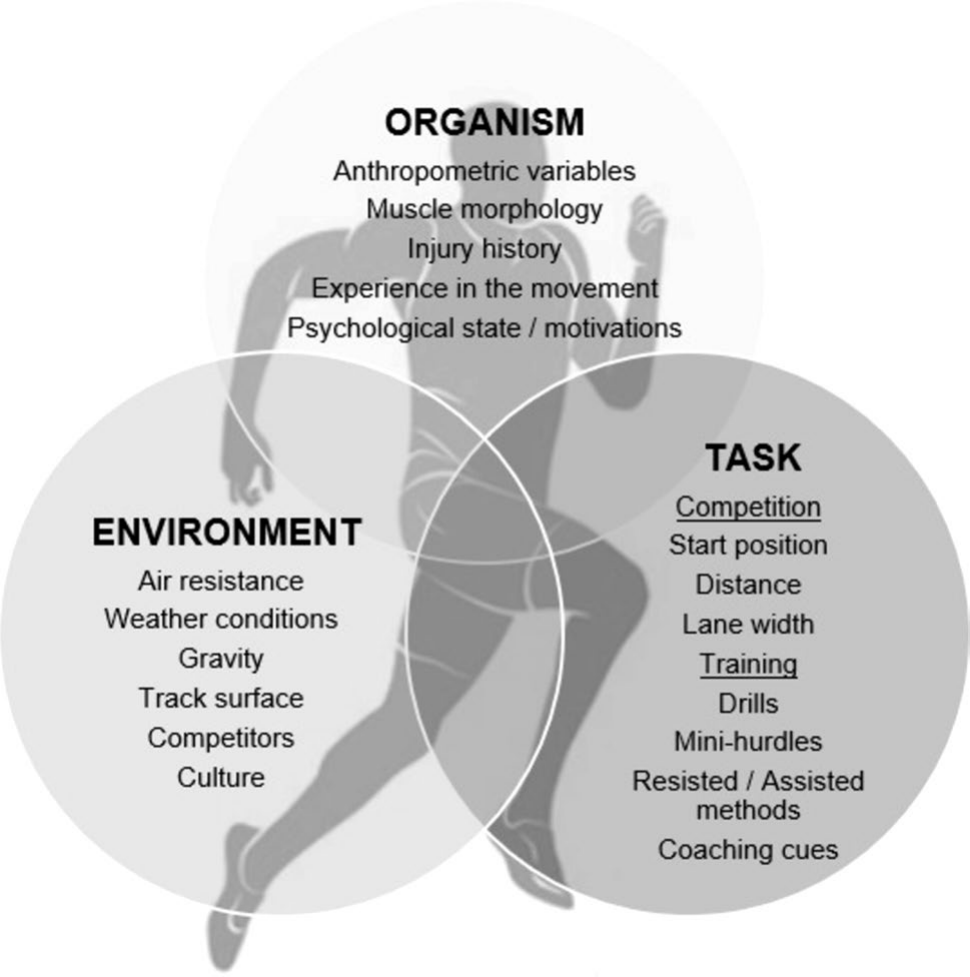
Schematic of the sources of constraints and boundary conditions to action within sprint running, which must be considered from the perspective of the athlete, rather than at the level of the description of the source

Newell [73] divided the constraints and boundary conditions that had previously characterised motor development approaches into three broad categories: (1) organism; (2) environment; and (3) task.^3^ The extent to which the chosen designation of all categories as constraints is due to the simplification of the terminology or reflects the latent attachment to the necessity of pedagogical guidance through constraining interventions cannot be clarified on the basis of the text. However, the consideration of organismic or environmental conditions as constraints and not as abundant possibilities or more neutral boundary conditions points in this direction. Either way, this framework provides a coarse suitable foundation to understand how coordination patterns emerge during all human movement and specifically sprinting. Boundary conditions can be considered both internal and external factors that interact through tasks to shape the systems’ biomechanical degrees of freedom and therefore movement and include anthropometric variables, coaching cues and weather conditions, Fig. 2 [38, 74]. Additionally, the boundary conditions of the athlete system can reduce or create alternative properties or possibilities for components to emerge owing to the relationship with the system’s conditions [75]. Sprint athletes constantly navigate boundary conditions via their own intrinsic dynamics, such as preferred mode of coordination or previous movement experiences, which research suggests is highly individual [76]. For example, a sprinter with long limb segments may leverage a greater step length at maximal velocity [77] but must therefore coordinate larger inertial demands at the hip as a trade-off [78].

Among categories, organism boundary conditions are particularly important for shaping sprint coordination and performance. These conditions refer to the intrinsic characteristics of the athlete and can be divided into structural boundary conditions, for example, height, mass, limb length and functional boundary conditions, for example, motivation, emotions and fatigue. A notable example is Usain Bolt, world-record holder in the 100-m and 200-m events, is listed at a height of 1.96 m (6′4″), with a mean step length of 2.44 m during his world record (100-m) performance in the 2009 World Athletics Championships [79]. Bolt’s competitors in the same race displayed a mean step length of 2.22 m; a 22-cm deficit per step [79] that had to be compensated by a higher step frequency. Compared to his peers, this structural boundary condition allowed Bolt to take almost four fewer steps across the race (*n* = 41.00 vs 44.91). Notwithstanding, it is important to point out that this does not imply a simple trade-off between step length and step frequency; many tall athletes do not achieve sprinting success. Along with step length, individual structural boundary conditions such as foot–ground interactions and muscle–tendon properties [80] have also been proposed as characteristics shaping sprint performances of the Australian junior sprinter, Gout Gout [81]. Bolt and Gout’s ability to coordinate greater step lengths with exceptional neuromuscular output and control illustrates how structural and functional boundary conditions interact to produce elite sprint performance.

Task constraints complement individual boundary conditions by defining the rules governing the task and include specific performance goals, equipment and information (e.g. verbal descriptions, physical demonstrations) provided by the coach. Environmental boundary conditions refer to physical and sociocultural influences that shape how an individual interacts with their environment including weather, gravity, ambient light, temperature, ground reaction forces and culture. Finally, the concepts of timescales in boundary conditions and constraints [82] highlight that some conditions change slowly if at all (e.g. anthropometric variables) while others fluctuate rapidly (e.g. fatigue). Understanding the time scales of boundary conditions and their influence on system behaviour reveal what is termed a nested organisation of boundary conditions across different levels, which has practical implications for coaching [82]. For example, rapidly changing conditions such as fatigue have short-term effects on sprint coordination, whereas slower changing conditions will influence movement patterns over longer periods, guiding both the timing and type of instruction provided by the coach [82]. Across sprint phases, horizontal force production can be classified a short-term boundary condition that fluctuates with neuromuscular readiness or fatigue [83], yet strongly influences the ability to maximise forward propulsion while accelerating [84]. In contrast, developing the capacity to produce greater levels of force and direct it horizontally, key to acceleration [85], occurs over longer time scales through training adaptations, therefore acting as a slow-changing boundary condition underpinning sprint performance. By manipulating and guiding constraints, practitioners can shape each athlete’s learning environment to allow individualised coordination and control patterns to emerge within system boundary conditions [86].

## Coordination and Control in Sprinting

Building upon the interaction of boundary conditions and degrees of freedom, further research suggests sprint performance relies on whole-body kinematic and neuromuscular coordination and control strategies [14, 87]. This contrasts reductionist models that attribute sprint performance solely to individual components, such as isolated kinetic variables, for example ground reaction forces. Bernstein [45] proposed coordination reflects the organisation of the motor apparatus (e.g. the central nervous system), and can be  considered the outcome of control. In sprinting, coordination can therefore be seen in the relative timing and sequencing of limb movements during the stride cycle, that results from the control that pertains to the magnitude and velocity of joint rotations and force application.

**Fig. 3:**
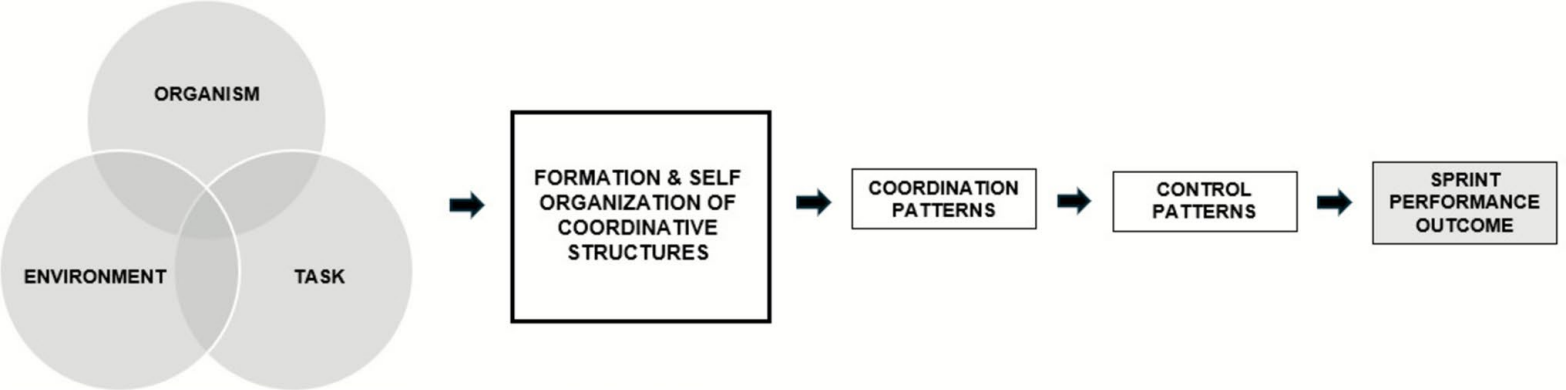
Theoretical model describing the interaction of constraints and boundary conditions and the role coordinative structures play in developing coordination patterns and the performance outcome. Adapted from Glazier [52]

Expanding on these ideas and drawing from Newell’s interacting model [73], Glazier [52] proposed a theoretical model within the dynamical systems framework, Fig. 3, illustrating how the confluence of boundary conditions (acting on a system) shape the formation and self-organisation of coordinative structures, described as task-specific ensembles of muscles [88]. Within a sprinting context, these structures give rise to observable coordination and control patterns such as step kinematics, limb segment sequencing and force application at different phases of the sprint, which collectively determine performance outcomes. The progression from boundary conditions to outcome highlight that sprint performance relies on the dynamic integration of several factors, with their relative influence constantly shifting over time. Importantly, it is worth noting (as Glazier has), the position and degree of overlap between the three constraint and boundary condition category circles can shift: the Venn representation capturing the dynamic nature of boundary conditions and their changing influence on performance at any given moment.

Furthermore, Lashley’s concept of ‘motor equivalence’ and Bernstein’s concept of ‘motor redundancy’ suggest the brain may preferentially select specific coordinative structures based on system conditions, allowing adaptability under changing boundary conditions [36, 89]. Similarly, Kelso et al. [34, 90] described coordination with reference to phase transition (e.g. a spontaneous re-organisation of movement patterns due to changes in a control parameter such as velocity), stability and flexibility of movement, and movement variability, all of which underpin athlete-specific movement.

During sprinting, the athlete faces the movement ‘problem’ of how to achieve higher velocities via improved limb segment coordination [24], 91, 92 and producing the corresponding forces at the right moment. While understanding segment organisation has been proposed as central to sprint technique, most research has focussed on quantifying dynamic variables such as horizontally and vertically directed force production, joint torques, impulses and spatio-temporal kinematics during different phases of the sprint action [7, 93–100]. These are often presented as determinants of performance for acceleration or maximal velocity, yet they provide only a first coarse approach into the underlying coordination and control patterns that produce these outcomes.

**Fig. 4:**
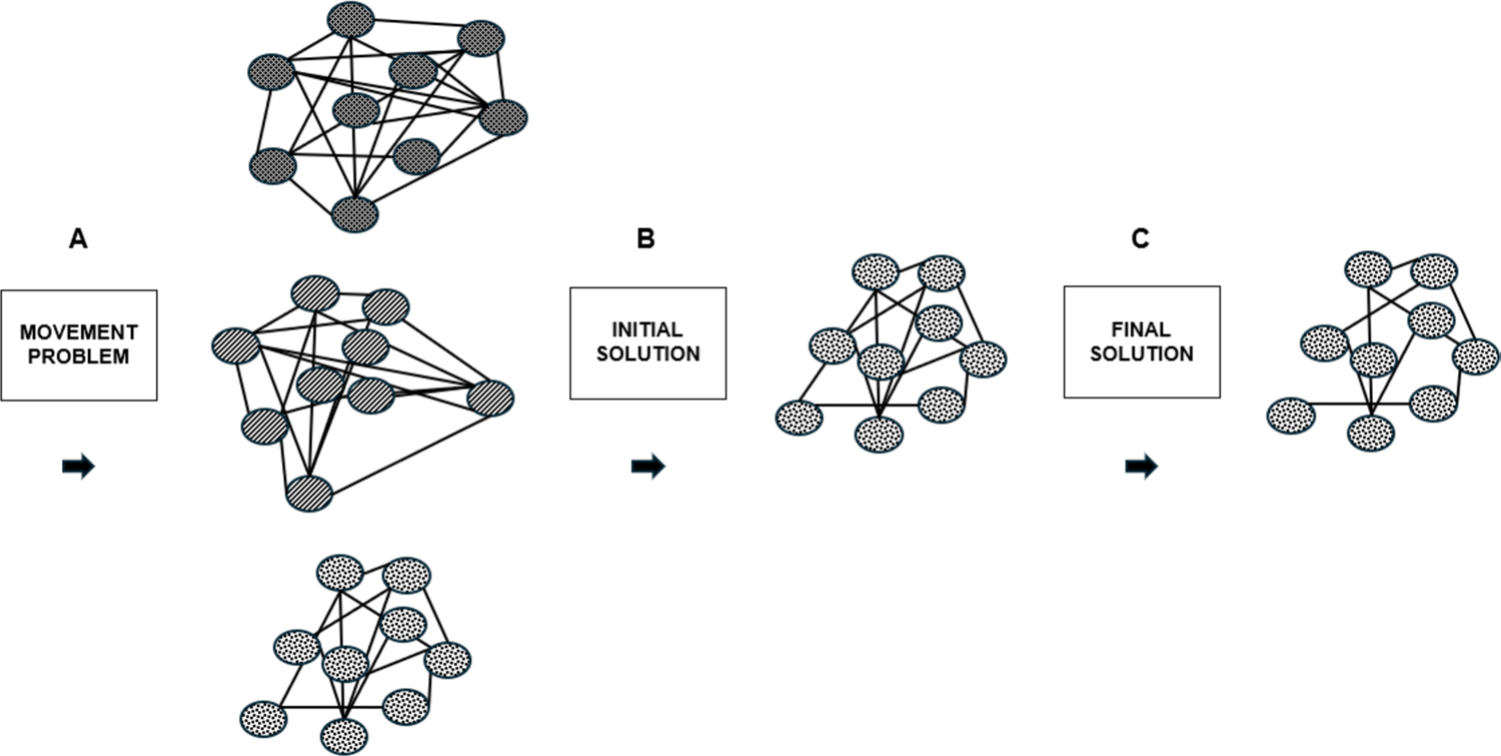
**A** The system must self-organise and select from a range of coordination patterns (with different degrees of freedom) that can solve the movement problem. **B** The selected coordination pattern operates within the available mechanical degrees of freedom, constraints and boundary conditions of the system.** C** The system has self-organised into a more efficient coordination pattern by, in this instance (not always), reducing (freezing) degrees of freedom. (Nodes conceptually represent body system joints. Links between nodes represent available degrees of freedom)

To address this gap, researchers have examined the relative movements of these system components using a continuous relative phase analysis [101, 102], vector coding, coupling-angle diagrams [24, 103–107] and pattern recognition approaches [78, 108]. These approaches quantify inter- and intra-limb coordination patterns, along with within and between athlete movement variability [32] and begin to uncover organisational strategies underpinning force production and technical execution while sprinting. In this context, phase relationships between body segments can serve as potential order parameters, describing the macroscopic essential features of a coordination pattern, as has previously been explored in gait studies [32, 109]. Importantly, seminal works of Bernstein and Turvey on coordination appear to identify how active mechanical degrees of freedom provide several coordination patterns to solve a single movement problem [36, 45]. Figure 4 illustrates schematically, various stages of coordination of a complex adaptive system may transit during a learning process. Interestingly, the question of the transitions between the individual states remained unresolved. It was only with the transfer of a phenomenon of DST, namely the increase in fluctuations during transitions from one stable state to another (69), to gross motor learning processes within the framework of differential learning (DL) theory (31, 40) that the phenomenon of self-organisation became accessible to the training processes in sports. By increasing variability by stochastic perturbations, the athlete could then traverse a range of coordination patterns under the given task constraints to solve a movement problem (e.g. accelerating from starting blocks), then select a momentary solution based on individual boundary conditions, with specific different degrees of freedom. The coordination patterns can further adapt and transition via further self-organising processes following increased fluctuations due to constant freezing or freeing of mechanical degrees of freedom [45, 90, 109–111]. From this perspective, sprinting should not be understood through a single, fixed technical model, but rather as a dynamic process in which athletes continually re-organise coordination patterns to meet organism constraints and task demand.

Within a DST framework, the degree of movement variability in sprint coordination patterns presents flexibility in the ‘athlete-system’ to search for the appropriate and adaptable sprint solution and may be preferable compared to a traditional approach of absolute invariance in the movement execution through repetition [32, 45, 112]. Dynamical systems theory views movement variability as a necessary non-linear component to create coordination change [71], contrasting traditional views of variability that emphasise eliminating these errors via rote repetition [113]. Importantly, even in repeated practice, task variability is typically constrained within functional bounds, allowing athletes to maintain consistency where requested, while still permitting movement exploration. For example, when learning the skill of sprinting, high variability and system instability are naturally present and expected. Rather than eliminating this variability, the sprint practitioner should encourage self-organisation by increasing variability to elicit coordination changes to emerge over time. It is suggested quantifying coordination variability using these methods, may be the only ‘true’ method in which a practitioner may fully consider the emergence of new coordination patterns [24].

### Coordination Classification

A coordination pattern classification scheme, as described by Chang et al. [114] and Needham et al. [104], has recently been explored in sprint research [24]. This technique builds upon vector coding techniques and describes the coupling angle as the vector orientation observed between two adjacent time points on an angle-angle diagram in reference to the right horizontal; expressed as angles between 0°and 360° [114]. The spatio-temporal focus of this approach can be preferred over other methodologies, e.g. range of motion analysis, static joint comparison, as it can allow more differentiated interpretation of both variability and movement coordination [115]. As most sprint coaching literature emphasises technical models focussed on shapes, positions, and segmental relationships (e.g. torso, thigh [hip-knee] and shank [knee-ankle]) at key phases of the sprint effort [6], quantifying inter-segmental relationships provides another level of analysis for the practitioner.

**Fig. 5:**
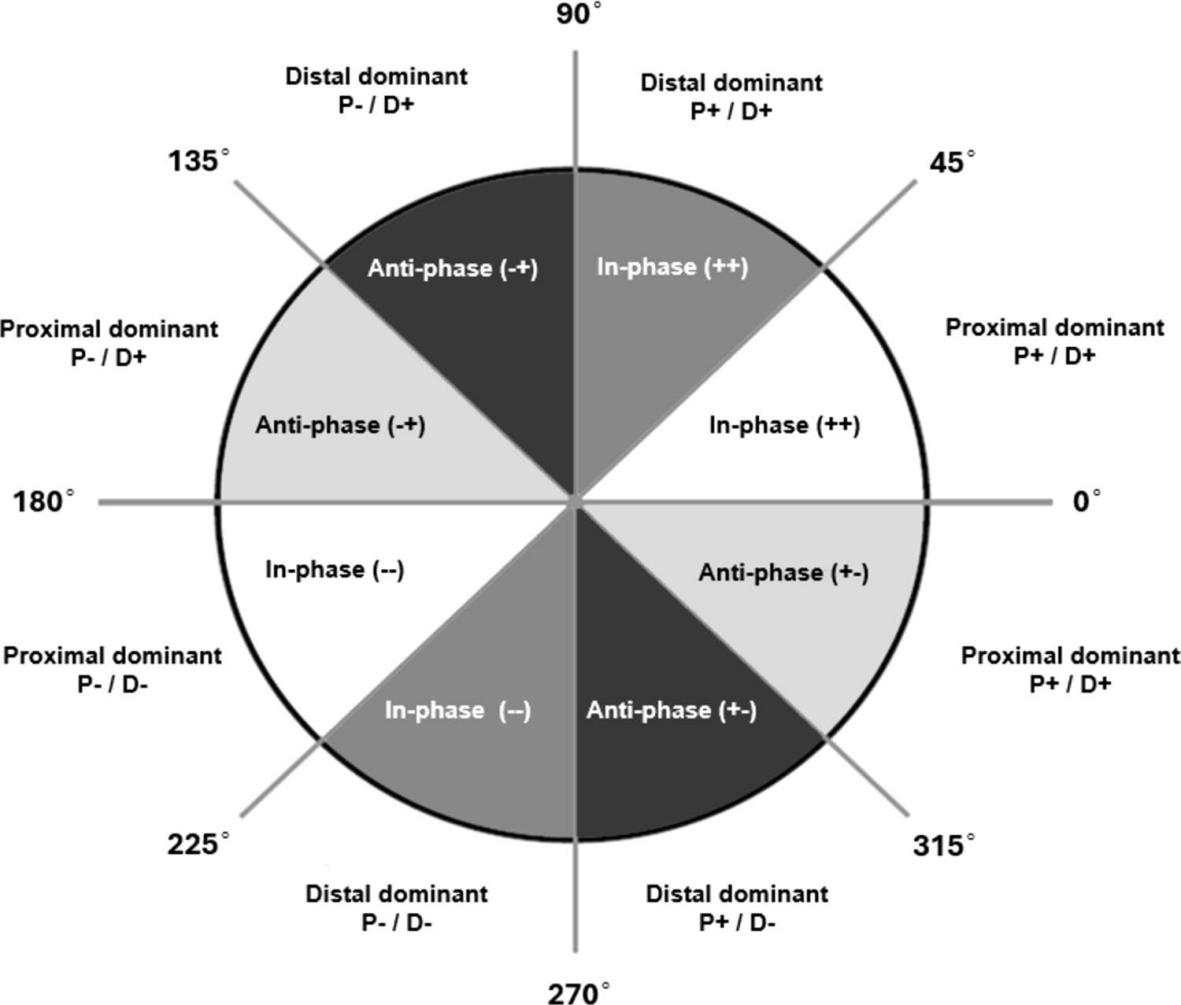
Coordination pattern classification system with coupling angles divided into eight coordination bins. Segmental dominancy (%) is shown around the circumference of the polar plot (P = proximal/*x*-axis, D = distal/*y*-axis). Adapted from Needham et al. [103]

To quantify specific coordination patterns, coupling angles between segments are divided into eight 45° coordination patterns (described as coordination bins), classifying coordination as in-phase (two segments rotate in the same direction) or anti-phase (two segments rotate in opposite directions), with either proximal or distal segmental dominance, see Fig. 5 [104]. Quantifying coordination patterns and their individual variability is reported to be useful in defining meaningful longitudinal changes, where changes outside of this variability would be considered ‘true’ modifications to sprint coordination [24]. In this instance, segmental dominance refers to a greater change in the angular range of either the proximal or distal limb segment at each timepoint during a movement cycle, for example, a stride [103]. Identifying which segment dominates provides insight into how athletes coordinate sprint actions across the kinetic chain, offering practitioners a more differentiated understanding of whether propulsion or control is being driven more by proximal or distal contributions at different sprint phases.

Research using this approach in sprinting has primarily focused on intra-limb coordination [101] (e.g. hip-knee or knee-ankle within a single leg) and inter-limb coordination [23] (e.g. thigh-thigh or trunk-shank across both legs). Intra-limb analyses highlight how individual joints within a limb interact to generate amplitude and direction of force and maintain stability, while inter-limb coordination examines how the limbs synchronise to optimise stride mechanics and efficiency. Additionally, this approach to coordination analysis highlights specific insights that may inform future training interventions across sprint phases.

### Acceleration

Coordination during the acceleration phase of sprinting has previously been explored in elite and world class sprinters (*n* = 21) to analyse technical differences in performance [106]. Using a vector coding approach [114], inter-limb (thigh-thigh) and intra-limb (shank-foot) coordination was determined from lead and trail thigh angles and lead limb shank and foot angles in steps 1–4. Applying a hierarchical cluster analysis, distinct lower-limb coordination strategies were evident across the initial steps [106]. Using examples linked to coaching implications, key differences included timing of limb reversal in thigh rotation, which can be classified as a ‘swing-leg retraction strategy’ [106, 116]. Longer in-phase clockwise rotation was also noted at toe-off, therefore delaying swing leg recovery, which in applied practice is linked to ‘over-pushing’. During the stance phase, one performance cluster exhibited more shank dominant anti-phase dorsiflexion; therefore, highlighting a potential reliance on using a ‘shin roll’; a strategy used to move the centre of mass forward and also identifies the orientation of force into the ground [117]. This type of cluster analysis, within a heterogenous population, further illustrate that individual coordination strategies can systematically diverge from a commonly cited sprint technique.

Similarly, acceleration capabilities were quantified in highly trained sprinters to determine between-individual variation and step-to-step changes in coordination [21]. Within this population, thigh-thigh coordination was largely anti-phase dominant and trailing thigh dominant. Variation in thigh-thigh coordination was greatest during block clearance and toe-off, thereby altering the timing and effectiveness of the ‘scissor action’, a key technical feature noted in sprint coaching [118]. When comparing trunk-shank coordination, this pattern was largely shank dominant (e.g. greater reliance on shank for propulsion) during the stance phase, with both segments working together to influence force production and centre of mass position at toe-off [21]. Coordination between these segments differed the most prior to and during the stance phase, likely due to varying individual mechanical degrees of freedom. Several shank-foot coordination patterns were also evident across participants. Key to this coordination pattern was foot dominant dorsi-flexion (anti-phase) during early stance, with brief in-phase shank periods before and after the stance phase. Greater anti-phase foot dominance was noted in step 1 compared with later steps, potentially highlighting strategies resulting from individually imposed constraints to complete the task.

A further comparative study focussed on elite and sub-elite coordination patterns during the acceleration phase used a coordination classification approach to determine technical differences between groups [23]. Across the first four steps, elite athletes demonstrated greater anti-phase, thigh-thigh dominance, compared to their sub-elite counterparts, for example, stronger ability to separate thighs in opposite directions at the same time (one driving forward while the other drives back), compared with less experienced athletes. Closer analysis of the thigh-thigh coupling angles highlighted sub-elite athletes spent a greater period in a trailing pattern near the time of touchdown, for example, slower swing thigh retraction, and in a leading pattern in the final part of the stance phase. Whereas this coordination pattern was not evident in the elite group. These findings provide empirical support for the coaching cue of ‘switching the limbs’ during initial acceleration, which may be key to maximising forward centre of mass propulsion in elite populations [119].

Collectively, this evidence indicates coordination during sprint acceleration is largely shaped by step-specific demands and individual athlete strategies, particularly in the timing of thigh retraction, involvement of lower limb segments in propulsion and force orientation in the initial steps. These coordination patterns reinforce the notion that the initial steps of a sprint depend on how athletes manage the interplay of these acceleration-specific constraints. Recognising these differences is important for practitioners as it highlight the individualisation required to promote true coordination change.

### Maximal Velocity

Changes to inter and intra-limb coordination patterns have also been observed in sprint athletes at maximal velocity, specifically in response to training interventions. Lenthall et al. [24] quantified lower-limb coordination profiles at maximal velocity to highlight differences in coordination strategies following a 6-week multicomponent training intervention and reported shifts in swing-phase strategies relative to ground contact time. These changes lead to reduced proportion of stride spent in anti-phase (ipsilateral thigh dominance), highlighting a greater contribution of the contralateral limb to developing a new coordination pattern. Similar findings suggest thigh-thigh coordination was mostly anti-phase across the stride action at maximal velocity, a coordination strategy commonly seen at higher running speeds [23, 102]. It was concluded this intervention shaped ‘swing phase’ mechanics, altered the point of ground contact and created more favourable vertical force transmission into the ground supporting the reorganisation of the mechanical degrees of freedom within a DST perspective.

In similar studies [101, 102], lower-limb coordination patterns were analysed during the stance and swing phase of athletes of different running speeds. Large between-group differences were reported in shank-shank and thigh-thigh coordination between the physically active (slower) and track and field group (faster), with greater anti-phase patterns at maximal velocity in the faster group [102]. Because of brief ground contact and swing phase times, faster athletes used a type of ‘feed forward’ control, rather than feedback, further supporting the notion that sprint coordination is shaped by task-dependent features that depend on the individual possibilities, rather than solely movement speed [102]. Anti-phase motions at maximal velocity also identified [101] changes to lower limb coordination strategy upon touchdown (knee-ankle and hip-knee coupling angles), as the athlete transitioned from swing (open chain) to stance (closed chain) phase. These strategies appear to regulate the timing and phasing of limb segments to enhance force transmission within extremely brief contact and swing phases. Coordination changes between shank and thigh segments during stance were also proposed to inform running speed potential, as limb positioning impacts ground reaction force and reduces ground contact time.

Finally, contextual factors further influence these patterns. Incline sprint efforts (16.0% inclination) promoted a greater proportion of anti-phase kinematics, which may in fact benefit acceleration coordination patterns via improved leg switching or recovery mechanics [107]. At lower inclines (5–10.5%), coordination strategies appeared more like those observed at maximal velocity [107]. Additionally, despite the observed inter-athlete variability, fatigued sprint athletes mostly showed greater thigh-thigh coordination changes during the middle to late swing phase of their stride, and a decrease in coordination variability during the stance phase. This fatigue induced change suggests a freezing of their individual degrees of freedom, or alternative coordination strategies are selected to maintain position during force application [120, 121].

This body of evidence suggests while specific patterns (e.g. increased anti-phase thigh-thigh coordination) appear more consistently at higher speeds, the variability across conditions and individuals highlights that there is no single fixed “technique”. In turn, this supports a DST perspective that maximal velocity sprinting is not defined by universal movement templates, but by context-dependent coordination strategies that self-organise to meet individual performance demands.

### Coordination Strategies

Various studies have attempted to identify, profile and cluster athletes based upon their coordination strategies [22, 87, 106, 122]. For example, similar to Schöllhorn’s research [92], approaches to cluster step characteristics during the block start, acceleration and maximum speed have been explored based on kinematics and dynamics of high-level sprinters. Donaldson et al. [22] identified three coordination strategies in elite male sprint athletes that differed during early flight phase, early stance phase and at the onset of swing thigh retraction. Similarly, Wild et al. [87] used a cluster analysis to identify a ‘whole-body kinematic approach’, for example, a coordination strategy, in professional rugby union players to establish four sub-groups based on step kinematics. By clustering or grouping athletes based on their individual sprint coordination strategy, it provides practitioners with scope to design future training interventions to target the coordination strategy the athlete relies on to produce effective sprint performance. Additionally, this approach may facilitate changes to the individual coordination strategy by using various coaching cues or methods, to direct the athlete towards key markers (step length/step frequency, flight time/contact time) identified in the cluster process. The authors propose that a cluster approach may provide an individual monitoring tool to sprint performance, allowing coaches to explore a given performance from a more holistic perspective. Collectively, this growing body of research emphasises the complexity, individuality and situatedness of sprint coordination patterns observed during acceleration and maximal velocity, raising important questions about which coaching strategies support effective sprint coordination.

## Coordination in Sprint Coaching Literature

The sprint coaching literature typically contrasts a DST perspective on coordination instead focussing on a range of isolated running drills, exercises and movement patterns to encourage the development of optimal sprint coordination patterns [39, 42]. As discussed earlier in this review, a reductionist approach is often assumed by sprint coaches, where isolated drills are prescribed that involve the athlete practicing a component of the sprint action, before attempting to implement the action into the entire sprint action, for example, whole-part-whole pedagogy [123]. This approach assumes refinement of isolated parts of the system will lead to enhanced sprint performance once ‘reassembled’ into an entire sprint effort. In line with this, expert sprint coaches have argued that dividing a sprint effort into distinct phases, acceleration and maximal velocity, is beneficial for coaching practice, as each phase has its own technical key performance indicators, position or shapes [3]. Furthermore, within a DST framework, we argue the concern is not that drills, or phase distinctions are inherently ineffective, but that the assumption of linear transfer from isolated practice to whole performance may overlook the complex processes of coordination, especially the transitions between the defined phases and the continuous variations over time. The varying relationship between individual hip extensor muscle size and activation and sprint running performance indicate in the same direction [124, 125]. Furthermore, limited agreement exists in the coaching literature about exactly which ‘shapes’ or ‘positions’ should be taught by the sprint coach (and in what context), and whether this information is supported in the research.

Morin and McMillan [3] have suggested athletes have an individual movement signature, yet specific kinematic positions or shapes are necessary to enhance sprint performance. During acceleration, the literature suggests ‘forcefully extending the stance leg’, ‘swinging a flexed thigh, forward and upward’, and ‘flexing and extending the arms to counterbalance the legs’ [3] are functional anchor points of the coordination pattern. Further, Mann and Murphy [126] extended on the position of ‘swinging the leg forward’ and discuss the concept of ‘front-side mechanics’. Simply, by drawing a vertical line down the middle of an athlete’s trunk (in the sagittal plane), when in an upright position; limb segments in front of this line are termed ‘front-side’ and limb segments behind this line termed ‘back-side’ [100]. Elite athletes are frequently described as minimising backside mechanics and enhancing front-side mechanics, as ground reaction force is negligible during the latter part of stance, for example, immediately before toe-off [126].

However, this mechanical strategy is not universally applicable. Evidence suggests variability in ground contact signatures among developing sprinters [17], one that emphasises greater vertical impulse during the second half of stance, rather than the first. For these athletes, force production and impulse generation are heavily reliant on the latter part of ground contact. We argue that an attempt to shift toward an ‘elite’ strategy too early, by minimising backside mechanics, can disrupt force application and impair sprint performance by ignoring the reality of varying physical capacities and coordination strategies.

This difference is reflected in how the sprinting task is modelled. Sub-elite sprinters (e.g. college athletes) [17] typically display movement patterns consistent with a spring-mass model [127, 128], where the body is treated as a single mass supported by a spring-like leg, and force is distributed more evenly across the full ground contact phase. In contrast, elite sprinters demonstrate patterns more aligned with what Clark and colleagues [129] have described as a ‘two-mass model’, where an early frontside-dominant application of force reflects an active repositioning of the swing leg relative to a stable forward-projecting pelvis. This model recognises the dynamic interaction between the lower limb and the axial mass in a way the spring-mass model does not fully capture.

That said, this distinction is not a barrier to development. While sub-elite athletes may initially rely more on a spring-mass strategy [17], it is reasonable to suggest that through interventions targeting for example, improved stiffness at ground contact, or swing-leg retraction [116], elite athletes may gradually shift toward a more frontside-dominant coordination pattern, two-mass strategy as their physical capacity evolves and boundary conditions allow. However, we argue this shift should be seen as a product of physical and technical development, not as an early technical cue forced onto athletes before they can express it effectively. Determining readiness involves observing whether the athlete can reliably express coordination patterns that are effective, sustainable and translate to sprint performance.

Clark et al. [118] also investigated the kinematics of rapidly extending the thigh from the ‘front-side’ of the body, which was termed ‘whip from the hip’. This coaching cue emphasised an exchange of limbs, for example, a thigh scissor action from front to back [130], thought to be useful because of the relationship with vertical force production upon ground contact. Despite this common coaching cue, Haugen et al. [100] did not find significant relationships between ‘front-side’ mechanics and maximal velocity sprint performance. This apparent disconnect further highlights a gap between coaching practice and the research, supporting the notion individual boundary conditions of the system likely shape the movement signature for each athlete.

In a further review on sprint performance and coaching [4], sprint-specific isolated movement patterns such as hurdle drills, walking high knees, running high knees and straight leg bounding were recommended to be used to teach various technical components of sprinting. Performed initially at low speeds and then progressing to higher speeds, the drills are aimed at ensuring a progression to normal sprinting. Whelan et al. [1] interviewed 209 sprint coaches about the use of sprint coaching drills to develop optimal movement and coordination and reported specificity to normal sprinting was key. Drills such as the A-skip, A-march, A-run and heel flicks were identified as the most used, as they phenomenologically represent ‘sprint technique’. Despite this link to technique, the authors noted coaches typically based drill selection on what was observed of more successful coaches, regardless of why it was performed.

As tempting and as traditional as it is to rely on the phenomenological similarity of exercises to the target movement, or more recently demanded as representativeness, this approach is constraining and misleading. A comparison of the kinematic and dynamic affinity [78] between a running stride in a 100-m sprint and four selected training exercises (five-step hops, two-legged hurdle jumps, drop jumps, 1/2 squats with load) showed similarities in joint force and joint moment curves for the drop jump movement, for example, but only in the vertical direction and with significantly lower amplitudes than in the maximum sprint. Surprisingly, the exercise with the greatest phenomenological similarity, the five-step hop, showed very little correlation in the hip joint force and moment curves. Furthermore, despite this lack of affinity, all exercises are considered effective in practice. The extent to which these deviations are prerequisites for neuromotor adaptation has yet to be conclusively clarified, but from the perspective of increased fluctuations in the DST, they are a logical consequence at the muscular and neural level. From this perspective, biomechanical diagnostics offers very meaningful tools but does not claim to know how the diagnosed coordination patterns can be changed.

Similarly, a muscle-functional biomechanical analysis of the classically executed heel flick exercise leads to problems with its similarity to the sprinting movement. From a biomechanical point of view, the heel flick fulfils two functions. First, heel flicks bring masses (lower leg and foot) closer to the centre of rotation (hip joint), which reduces the moment of inertia of the leg and increases the speed of hip flexion. Second, the rectus femoris muscle, as the only two-joint muscle part of the quadriceps femoris, supports hip flexion, but can only do so optimally if it is pre-tensioned by an overextended hip and simultaneously bent knee, as happens at the end of the support phase. However, if the heel is only brought to the buttocks with the hip flexed, this effect is lost. The instruction should therefore not be ‘heels to the buttocks’ but ‘heels to the shoulders’ in order to achieve pre-stretching of the rectus femoris muscle across both joints and thus make hip flexion more effective [39].

A further study using expert coaching perspectives [131] focussing on ‘good sprint running technique’ identified posture, hip position, ground contact and arm action as key movements patterns to sprinting. A similar study [42] analysing the technical features of sprint performance identified hip position, leg extension and posture as important movement patterns to observe during the action. Yet, the description provided limited information of how to achieve these actions and patterns, nor their relationship with sprint coordination and performance [78].

One coaching practice suggested to enhance sprint coordination patterns are ‘dribbles’. Goodwin et al. [132] have described a ‘dribble’ as a miniature sprint, characterised with the key technical patterns of usual sprinting, albeit at reduced running velocity and limb amplitudes. Compared to traditional sprint drills listed above, dribbles appear to replicate typical sprint coordination patterns such as limb timing, lower limb tension prior to ground contact, and promote earlier recovery of the swing leg, while also practiced as a ‘whole’ movement rather than the isolated form of most sprint drills, thereby reinforcing the coordination patterns and positions desirable during sprinting.

Finally, there is no consensus in sprint coaching literature on which drills coaches should implement to improve sprint coordination [131]. However, it appears sprint training methods, such as wicket runs, resisted and assisted sprint training, and wearable resistance [3, 5, 27, 28, 122, 133–136], attempt to provide a link between technical features, sprint coordination and performance. Essentially, these methods provide a task constraint for the performer, where the body must find an individual movement solution to the problem in front of them. While self-organisation in response to task constraints occurs across many movement skills, in sprinting, these drills encourage the system toward exploring more effective coordination patterns and in parallel make them more stable against eventual disturbances. One might argue, this is the case for all sprint drills, for example A-skips; however, the relevance of a drill becomes limited if it cannot meaningfully distinguish between targeted coordination patterns that actually impact sprint outcomes.

Most importantly, here two different forms of self-organisation on different time scales must be distinguished: self-organisation within a task (micro-level, associated with classically individual adaptation) and self-organisation across training (macro-level, associated with DST based increased fluctuations as in DL). In the first case, the athlete spontaneously ‘fixes’ the available degrees of freedom in a stable pattern in order to solve a single movement requirement under the current conditions. Couplings, relative phases and synergies arise spontaneously and stabilise or reconfigure the pattern (e.g. freezing/releasing degrees of freedom) without any external specification of how the movement is to be performed. In the second case, exploration across many different tasks or exercises reshapes the solution landscape, resulting in a new, more effective coordination pattern. Here, the athlete organises both each exercise and, cumulatively, the repertoire itself—variability drives reorganisation rather than simply optimising a fixed solution.

## Does Optimal Sprint Technique Exist?

When exploring the concept of ‘optimal sprint technique’, a DST perspective would suggest inherent movement variability, for example trial-to-trial variation, and functional degrees of freedom within the movement system, limits the reductionist explanation of one specific coordination pattern or ‘technique’ for a movement task [52, 72, 86]. Dynamical systems theory views movement variability as a necessary non-linear component to create coordination change that goes beyond Bernstein’s famous quote of ‘repetition without repetition’ [71, 137]. The DST point of view also contrasts traditional views of variability in movement patterns, where a focus is placed on eliminating these errors over time by refining one consistent movement pattern [113]. For example, when learning the skill of sprinting, high inter-trial variability and system instability are naturally present and expected, yet generally not related to optimal technique. While this variability is not directly indicative of an optimal technique, it may facilitate exploration of movement solutions that eventually converge on more effective sprint coordination patterns [24].

Hatze [138] suggests optimal technique consists of “ … motions yielding a maximal performance under given constraining conditions and for a given individual”. Similarly, Newell [73] describes optimal patterns of coordination and control in movement tasks as individual specific based on the interaction between constraints and boundary conditions. Despite the assumption of individually optimal patterns, there were no methods to prove them, and no answer was given as to how to proceed once this optimal technique had been achieved. Variability was primarily used on the way to a presumed optimal technique, but how to deal with it once the goal had been achieved remained an open question. From this perspective, coaching cues can be viewed as one type of constraint that interacts not only with the organism (i.e. the athlete), task and environment but also situative conditions, shaping but not determining a coordination solution [139]. In addition, the focus shifts from cueing athletes toward a single model of sprint technique to designing environments and instructions that facilitate individualised yet functional movement solutions [140].

Despite the DST perspective, the concept of an ‘optimising’ technique to enhance sprint performance is ubiquitous in the literature. Research has focussed on coaching cues and drills [1, 42, 141], step and sprint phase kinematics [11, 67, 142, 143], sprint-specific training methods [144, 145], mechanical characteristics during acceleration and maximal velocity [15, 68, 144, 146], assessment scores [147] and predictive simulated modelling [142, 148]. In these instances, kinematic variables are averaged across a group of athletes (e.g. elite), then used as a reference point or a criterion, upon which lower level athletes are compared against, effectively setting performance benchmarks [72, 149]. The thought process being, by averaging key variables, a common coordination pattern will emerge, which is optimal for each athlete undertaking the same movement task [52], thereby enhancing performance. For example, studies [79, 150] that have analysed performances from Usain Bolt, identified optimal step length and step frequency, thigh angular velocity and stance leg touchdown position as variables that describe ‘optimal sprinting technique’, with the inference this information could inform training prescription for other sprint athletes. World-class sprinters certainly differ from sprinters at national or local level in terms of typical biomechanical characteristics. The problem, however, is that within these groups, many individual solutions are applied. However, if the starting level is individual and the target level is also individual, then it becomes problematic to tell the athlete exactly in a reductionistic thinking which variable needs to be changed and how.

**Fig. 6:**
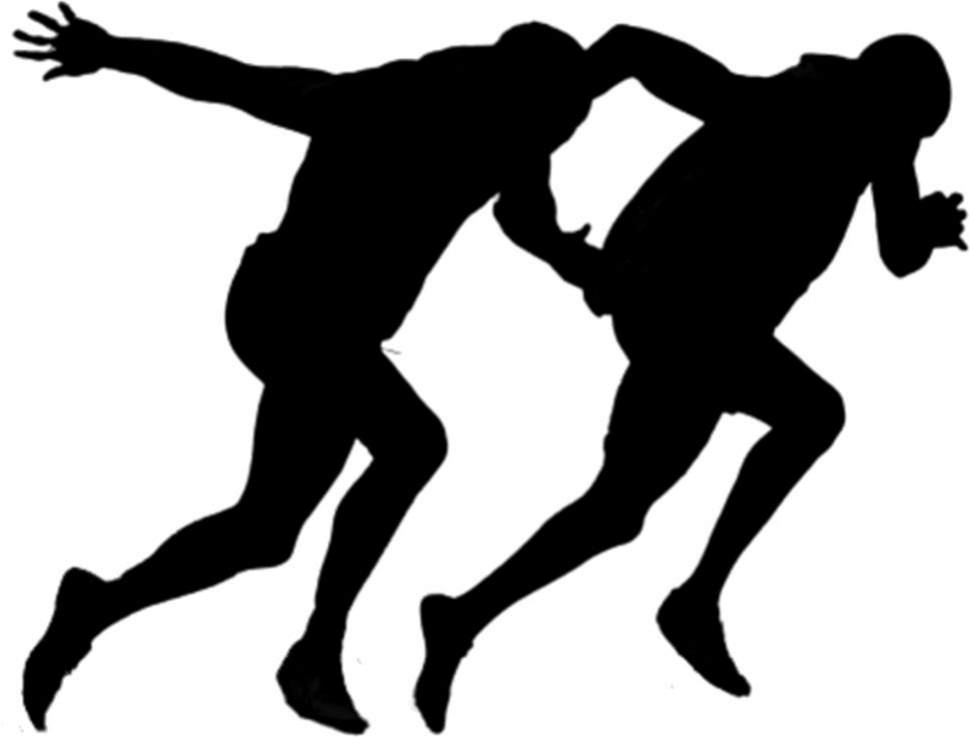
Two elite sprinters, each of whom have run below 20 s in the 200 m, displaying different coordination patterns at toe-off to achieve the same goal-directed task (e.g. block clearance into acceleration)

Further studies by Brisson et al. [151, 152] explored the concept of an ‘optimal movement pattern’ using different forms of feedback, for example, knowledge of results and performance, and suggested it was doubtful a common movement pattern for a given task exists. Moreover, despite recognising there are critical features necessary to achieve high performance in a motor skill such a sprinting, it was suggested there exists a kinematic continuum or movement bandwidth that displays differently for each athlete, rather than a specific set of variables that all athletes must achieve, Fig. 6. Additionally, the approach of forcing athletes to replicate a ‘gold standard’ ‘average’ coordination pattern, which may work in opposition to their morphological or physical constraints, is misguided.

Interestingly, a study that explored step frequency and step length in elite sprint athletes (*n* = 52, including nine athletes under 10.00 s for 100 m) concluded athletes tend to be reliant on a particular step variable to achieve their best sprint performance, thereby highlighting inter-athlete movement variability within performance [99]. In this instance, a group-level analysis may be an inferior approach to understand sprint performance, as it may mask individual athlete differences, and would not guarantee enhanced sprint performance. Similar studies on elite sprint athletes [153, 154] identified an individual reliance and relative importance on either step length or step frequency at specific phases of a sprint, further highlighting the variability exhibited in sprint variables and their importance to individual sprint success. Only when the step length and step frequency is normalised by the athlete’s leg length then a tendency toward overlapping principles can be seen [77]. One systematic review [7], focussed on the acceleration phase, suggested that as athletes have different physical and morphological characteristics, the optimal combination of step length and frequency should be addressed at the individual level; a conclusion also supported in rugby union players [87]. Further studies focussed in track and field (javelin) identified large variations in throwing coordination patterns in elite-level athletes, providing greater evidence against a common optimal movement pattern for a motor skill in a heterogeneous population [72]. Rather than negating the role of technical instruction, this perspective highlights the need for coaching instruction to align with organism constraints and boundary conditions. In this view, the athlete may not be able to imitate the technical representation provided by the coach, instead developing a movement solution that is conducive to their structural, functional and experiential boundary conditions [72]. This reframes ‘optimal’ not as a universal template, but as an individualised coordination strategy shaped by both coaching input and self-organisation.

Finally, when considering a complex movement such as sprinting, and the goal of the task is not isomorphic (e.g. the same) with the movement pattern, the evidence suggests there exists a range of coordination patterns to achieve the task based on an athlete’s internal and external boundary conditions and active mechanical degrees of freedom [151, 152, 155]. Notwithstanding, the process and explanation by which sprint coordination transitions between phases to a more preferred pattern for enhanced speed are limited through reductionist frameworks.

## Self-Organisation

Dynamical systems theory suggests an athlete’s executed movement originates in response the current constraints and boundary conditions on the system and the task at hand; a process referred to as self-organisation in two time scales. In a sprint context, this process occurs through increased variations in factors such as speed, force application or neuromuscular dynamics within the system’s boundary conditions. Such adjustments can influence the athlete’s posture, for example, transitioning from a more leaned-over position to a more upright sprinting posture, as part of an emergent movement strategy. Importantly, within a DST perspective, any movement solution can be classified as self-organising on the shorter time scale (micro-level) yet may not be inherently more or less efficient. Rather, the quality of the emergent movement pattern depends on how boundary conditions shape individual coordination strategies and whether these align with improved performance. For example, manipulation of constraints that guide the self-organisation process on the shorter time scale toward improved movement efficiency, orientation of antero-posterior or vertical ground reaction forces [66, 84], step length or step frequency [67] is supported in the research to align with the performance demands of sprinting. In contrast, a traditional skill acquisition perspective would frame these performance outcomes in terms of consciously processing single selected technical variables such as applying more force through the foot, limb exchange or ground contact time. At the elite level, where step frequencies could be as extreme as 4.5–5.1 Hz [99] and contact times as brief as 0.1 s, [67] it becomes difficult to justify such adjustments are the result of deliberate cognitive control, underscoring the role of short-term self-organisation.

Furthermore, self-organisation on the longer time scale (macro-level) offers the advantage of explaining how the motor control system transitions from an existing to a new movement pattern; by a process called phase transition [70]. In a sprint context, this would translate to the coach implementing variation in movement pattern(s) to influence how the athlete for example creates the ‘whip from the hip’. These variations would then be reinforced during subsequent training sessions, thereby attempting to alter the coordinative sprint pattern by shifting the variations from the geometric to the velocity or acceleration level (31). Phase transitions between stable movement patterns occur as emergent shifts in coordination patterns driven by control parameter changes, such as increasing velocity, rather than deliberate technical modifications made by the athlete or instructed by the coach. For example, as speed increases, athletes shift from walking to jogging, jogging to running, and running to sprinting. In sprinting, similar transitions occur between initial acceleration (terrestrial/ground bias [156] and major emphasis on horizontal force application), late acceleration (aerial biased, and continued emphasis on horizontal force application) and upright sprinting (minimise ground contact, emphasising mass-specific vertical force application) [19], where changes in ground contact time, flight time, limb positioning and force application emerge spontaneously based on task demands rather than explicit coaching interventions [157]. Therefore, phase transitions represent the reorganisation of movement dynamics as an existing attractor loses stability [158]. Important to note, classical control parameters can be increased and decreased as desired, which differs in real learning processes where emergence is associated with sustaining changes over time and cannot be turned back. Once a real learning process happened, the reduction in noise rarely allows the system to go back to old patterns. However, this also highlights an overlap between these processes and maturation and ageing processes.

Within a DST framework, attractors are described as a preferential stable state of coordination [34], where the system is drawn or attracted to particular possibilities of coordination [37]. Attractors have developed by the former practice history as stable regions within the broader coordination landscape, in which the system is comparatively less likely to leave because of reinforcing specific training over time. In sprinting, practitioners often refer to features such as including front-side mechanics [126], hip torque [96], greater anti-phase, thigh-thigh dominance [23] and lumbopelvic control at maximal velocity [159] as desirable to observe in specific sprint athletes. While these features are commonly highlighted owing to their association with enhanced sprint performance, considered in isolation they represent only partial indicators of sprint coordination dynamics rather than attractors themselves. Less stable sprint coordination patterns can also represent local minima, for example, youth athletes, who will display ongoing issues to consistently repeat preferred coordination patterns during the movement task.

**Fig. 7:**
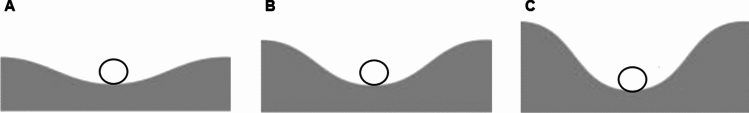
The ball in each image represents the state of the ‘system’. **A** A shallow attractor valley, where coordination patterns are less stable, requiring less perturbation to shift state. **B** A wide attractor valley that is stable and can tolerate perturbations yet also adaptable. **C** A deep attractor valley that is highly stable but less adaptable to change and would require significant perturbation to transition to a new attractor state

Despite changing an athlete’s sprint coordination pattern considered straightforward in principle (e.g. closed skill), the shift in attractor state, whether deemed minor or major is dependent on several factors. The current state (order parameter) of the attractor, the depth of the ‘valley’ (e.g. landscape) and the proximity of the proposed coordination pattern in reference to the existing pattern will determine the quality and effort required (control parameter) via a coaching intervention to induce a change, Fig. 7 [34, 160]. For example, when an athlete’s sprint coordination pattern during acceleration or maximal velocity is characterised by a deep attractor valley, it suggests the system displays high stability, and typically is understood to require more coordinative effort or energy to transition to a new valley (if this is the coaching focus) [33], thereby making it relatively resilient to minor perturbations delivered via coaching interventions. However, if we consider this two-dimensional form of representation as a significant simplification for a high-dimensional system, the effort involved is only considerable when moving within the dimensions described. However, if a dimension is found or opened up that is either not as stable or has been neglected up to now, a new attractor can be formed with little effort. Interestingly, these sensitive dimensions are usually individual specific (e.g. reflexes preserved from early childhood). Shallow attractor valleys highlight coordinative patterns that are less stable, thereby requiring less perturbation to shift state [33] and demonstrate lower adaptability and performance consistency. The depth of attractor valley may also describe the level of performer the coach is working with (advanced = deep, novice = shallow) but may also identify experienced sprinters who have been coached over a long period with specific technical cues, for example, they display certain stereotypes. Finally, the depth of attractor valley may also identify the non-linear approaches necessary to promote coordinative sprint changes across performance levels.

In seminal work on human locomotion, Diedrich and Warren [33] suggested there are certain characteristics of phase transition to new attractor states, which identify short- or long-term coordination changes. These include a re-organisation of the complex system, continuous changes in the control parameter (e.g. frequency or velocity of limb(s)), deviations between new and existing attractor states, decreased attractor stability and greater variability near the phase transition point [33, 161]. This could be illustrated by coaching an athlete towards a new attractor such as holding a neutral pelvis at maximal velocity (e.g. reduce braking forces on ground contact), or to limit over-pushing during acceleration (e.g. improved limb switching capability), yet the athlete consistently reverts to the existing pattern. These qualitative movement characteristics become important for the sprint practitioner to consider because their absence in a training environment may suggest the threshold to change attractor state has not yet been achieved and coordination changes are limited or null. Conversely, if phase transition characteristics are evident during the training process, it suggests the system parameters have experienced perturbations, for example, a spectrum of interventions to elicit phase transition and for the athlete to occupy a new attractor state [33]. For example, when analysing interventional studies [24, 159] where coordination changes are evident, it suggests phase transition has occurred as one attractor becomes unstable, and the system forms a new attractor state. These perturbation type transitions to new attractor states have been described as bifurcation or noise-induced tipping [162].

It is our assertion that not every intervention-based modification to sprint technique requires phase transition on topological level. Subtle modifications in sprint technique, for example in metrics, will occur within an existing attractor, rather than a complete destabilisation and shift to a new attractor. Many coaching interventions, particularly at the elite level, are focussed on stabilising and refining desirable coordination patterns, thereby creating a deeper (or wider) attractor valley. For example, coaches may place a continued focus on reducing extension of the trailing leg at maximal velocity, thereby providing greater time to reposition the lower limb prior to the next ground contact, leading to faster limb switching [163]. A focus such as this may only require small (rather than large) critical fluctuations to induce coordination changes and shift the system towards a transition point where the existing pattern becomes more stable [33]. Recognising when targeted coaching interventions should either reinforce or challenge existing coordination patterns is therefore essential knowledge. Finally, modifications to sprint coordination patterns must align with the intervention and skill acquisition approach, to either reinforce an attractor or induce a transition to a new coordination pattern, for example a new attractor.

## Applying Skill Acquisition Theory to Sprinting

Despite the critical importance of establishing stable sprint coordination patterns, there remains limited guidance in sprint coaching pedagogy regarding the skill acquisition theory or approach, which underpins how sprint coordination patterns are modified. As mentioned in this review, there is an extensive amount of information on the ‘what’ and the ‘why’ certain sprint coaching methods and techniques should be used; however, a significant gap exists about ‘how’ practitioners deliver this information to the athlete. To this point, there is also conjecture on whether practitioners of all sports have sufficient knowledge of skill learning theories and principles when constructing their sport session and whether these two variables align [164]. Unfortunately, the processes of acquisition, stabilisation and improvement of skills are still too often treated indiscriminately and generalised regardless of their origin, even though all theories to date have only referred to individual aspects of these processes [165].

Typically, skill acquisition in sprint coaching is presented from a cognitive psychology information-processing perspective [1, 5, 25, 26, 30, 123, 134, 135], where explicit instruction from the coach, drill, repetition and mental representations drives sprint coordination. This approach is based on first-order cybernetics with its objective information, which completely disregards the learner’s individual characteristics [166]. With second-order cybernetics, these characteristics became the focus of attention, as it was subjective information that was crucial for the learner’s progress. This depends on the learner’s prior knowledge and experience [165]). Dynamical systems theory provides a skill acquisition perspective to elicit sprint coordination changes considering more the individual specificities of the athlete by the process of self-organisation on the macro-scale. Schöner [167] suggests understanding coordination changes requires not only knowledge of information processing to compute the pattern, but also anticipating how the pattern is likely to evolve under various environmental and situational conditions established in the learning process. That is, the sprint practitioner may intend to change an athlete’s sprint technique, but various constraints or boundary conditions may limit whether the coordination changes ever emerge.

There are different pedagogical approaches that relate to the DST. The most known in English- speaking countries is the constraints-led approach, which is a framework where known pedagogical interventions have been systematised by alterations of the individual, task and environment [73]. Since its original introduction, this approach has experienced numerous re-interpretations. In the original, Newell’s transfer of Lewin’s behavioural model, which considers behaviour as a function of person and environment, systematised existing developmental approaches as an alternative to the prescriptive approach that was pursued in connection with the repetitive and role model-oriented approaches [168–170]. Theoretically, the three classes of constraints were considered as commonalities of existing approaches by means of which the athletes were guided towards a certain optimal technique. Importantly, the constraints served to channel the learning system towards an intended target movement. In accordance with reform-pedagogcial principles, athletes are required to actively discover this movement solution themselves, the approach has often been described in terms of self-organisation mainly related to a micro-scale. However, it should be noted this interpretation does not fully align with all defining characteristics of self-organisation on the macro-scale as proposed in DST (e.g. order and control parameters described by Kelso [34]).

Despite briefly discussing internal and external boundary conditions in sprinting earlier in this review, this section focuses on the application of using constraints, as conditions to guide movement, during coaching sprint athletes. Key to adopting a constraints-led approach, as a subset and specific interpretation of a DL model, is to ensure the primary focus of the training session, for example, a goal-directed pattern, is not altered by too much inappropriate noise. For example, if the primary theme or focus is to enhance coordination patterns at maximal velocity, such as thigh-thigh coordination, any restriction that inhibits the athlete from reaching their top speed should be avoided, such as external resistance. Once the coach has selected the training theme, for example, acceleration, maximal velocity, step length or step frequency, the training environment can be carefully designed to encourage movement exploration and practice variability in the narrow surrounding of an assumed ideal movement technique, allowing the athlete to self-organise within these constraints.

Mini-hurdles (or ‘wickets’) are an example of how a coach can manipulate task constraints to shape sprint-specific movement solutions. By adjusting the spacing, the coach can emphasise different aspects of sprinting. Placing the hurdles closer together, for example, encourages a higher step frequency, while spacing them farther apart promotes an increased step length. If neither the step length nor step frequency is the primary focus, varying hurdle distances within the training session can still be useful in introducing perturbations that challenge the athlete’s sprinting gait, requiring continuous adjustments in coordination, limb repositioning and force application. In this way, mini-hurdles act as task constraint where the hurdle distances are adjusted to the subjective information in terms of individual needs, guiding the athlete toward externally focused movement adjustments. Rather than instructing the athlete with internal cues like ‘pick your knees up’ or ‘hit the ground with more force’, which may disrupt self-organisation, the mini-hurdles create an environment where the athlete implicitly adapts their mechanics. This indirectly may lead to picking the knees (shorter hurdle distance) up or hitting the ground with more force (longer hurdle distance). If the open task is solved by leaning the trunk forward or backward, additional creativity of the coach is requested. Because of the individuality of the athlete not only in movements but also in communicating and thinking, variations in the style of communications shift the self-organisation to another level. In a similar context, studies in long jump [171, 172] found that practicing (e.g. jumping) in learning environments that require frequent athlete-task-environment modifications as an interpretation of increased fluctuations led to improved run-up consistency, take-off accuracy and step regulation (e.g. more adaptable athletes). Overall, these noise-driven variations may encourage the emergence of stable and efficient coordination patterns over time, reinforcing adaptability and robustness in sprinting without imposing a rigid technical model.

A more general learning approach, in which laboratory results on cyclic finger and limb movements in connection with DST were transferred to applied sports for the first time, is DL [31, 108]. Inspired by the own practical experiences as a high-performance athlete and coach of top athletes in various sports in the 1980 and 1990s, the parallel biomechanical identification of individual movement patterns in world-class athletes and the non-repeatability of identical movement patterns merged into the DL model [72]. The DL approach increases observed fluctuations to destabilise momentary stable movement patterns for initiating a self-organising process on the macro level. Thereby, a key aspect of the fluctuations is their inclusion into the principle of stochastic resonance. The diversity of exercises and an athlete’s variability are described as fluctuations that have to be attuned to each other (re-sonance) to achieve an individually optimal learning rate. In this context, every type of training intervention, regardless of whether it was caused internally or externally, is modelled as a certain amount and type of noise, thereby linking the principles of DST with second-order cybernetics and creating a model for athlete-centred training. Based on biomechanical, neurophysiological and pedagogical principles, DL emphasises increased variability and adaptability to new stimuli that occur even with each repetition.

In addition to ‘never train the right thing to become the best’ [173], the quote ‘by confronting athletes with a high number of practice activities, the probability increases that any of the training exercises can get in resonance with the athlete’s needs’ conveys the DL idea in a practical approach [173]. By offering continuously varying exercises, multiple situations are created in which the athlete gets the chance to immediately experience the consequences of the activities. Differential learning requires the practitioner to take advantage of the occurring fluctuations for learning and the reference point of the ‘to be learned movement’, which allow the system, for example, the athlete, to adapt to the continuously changing context [113, 174]. Instead of considering errors as something that must be avoided and reduced, DL considers deviations as the source for learning, the subjective information, and suggests the strategy of adding stochastic perturbations to cause larger differences between two subsequent movement for keeping the subjective information rate on a corresponding level. Including DL into the coaching process is accompanied with the highest learning rates of acquiring, stabilising and refining motor skills [71, 137, 175, 176].

Perturbations that have been used in previous DL studies in track and field focussed on variations in joint angles, joint velocities, joint acceleration and rhythm dependent on the level of the learners. Thereby, following a broad sequence of varying mainly movement geometry in beginners then progresses to varying dynamics, and finally advances to varying the rhythm of sprint-specific exercises [31, 174]. Depending on the ‘to-be-learned’ coordination pattern(s) the practitioner is trying to enhance, DL exercises may include altering start positions, running on different surfaces, exaggerating arm movements or specific stride patterns, varying the tempo or rhythm of a sprint or adding or removing external load as a constraint. It may also include a combination or contrast of several iterations of the exercise, creating endless number of variations. Despite these variations in movements appearing quite common in sprint training, it is the systematic approach by which these are implemented by the coach or by the athlete, and the intent to create an optimum amount of ‘noise’ in the learning process. While repetition without repetition is limited to variation of the movement metric, DL includes variation of the topology as well. While at the beginning of a learning process the variations are given by the coach, mainly to reintroduce the creativity in learning, in later phases, the athletes are encouraged to find other variations by themselves [31]. In addition to letting the athletes find their own optimal solution, the DL approach emphasises the training of adaptability [108], in effect allowing the system to exploit this abundance of solutions to achieve performance despite continuous variations of the boundary conditions on the micro-scale [177]. The adaptability training helps to cope with the impossibility of repeating a movement identically twice. This continuous change in movement patterns was supported by biomechanical studies on gait that could identify significant change in coordination patterns within brief periods without external intervention [140, 178].

In a 6-month sprint training study, comparing traditional training (× 5/week for 90 min) and a DL approach (differential sprint exercises, × 2/week for 90 min) [*n* = 15, personal best 11.4–12.8/100 m], pre-post assessments focussed on 30-m sprint tests measuring main joint angles, angular and centre of mass velocities plus ground reaction forces to determine group differences in regard to pedagogy [41]. The study reported the DL group improved their mean maximum velocity by 0.37 m/s, compared with the traditional sprint training group who improved by only 0.14 m/s [41]. The authors concluded that while the DL group improved their maximal sprint velocity to a greater level than the control group, individual coordination patterns and training frequency appear to have influenced the training effect. For example, athletes in the DL group were clustered in similar coordination patterns post-training compared with the control group, yet training content and duration were not volumed matched between groups [41]. Similar findings were observed in reference to maximal sprint velocity in a 6-week study focussed on youth sprint hurdlers [174], which found the DL approach had a significant effect on pre-post sprint hurdle performance, compared with the traditional repetition-oriented delivery of training. These findings identify DL as one skill acquisition approach to enhance sprint performance by leveraging and exploiting movement variability. Finally, studies on the effect of DL on brain activation as well as on the brain–heart interaction support the effectiveness in terms of producing frequencies that are associated with being advantageous for learning [179, 180].

A common aspect of these approaches is the coach modifying the task, to allow the athlete to engage in implicit learning to discover the best movement solution for themselves. In these approaches, analogy learning is often used where instructions such as ‘run like a cheetah’ can be used to let the athlete try to sprint differently, without letting the athlete know how to do it, [181]. Thereby, the athlete tries to solve the task implicitly. This has been found to be more effective on performance than explicit gained knowledge (information-processing perspective), when the athlete was put under pressure [182] as they do not have tacit knowledge about keeping the ‘right technique’. This leads to another approach that proposes learning without making errors [183] that may help athletes from ‘over-thinking’ technique during sprinting, as such a conscious focus may prohibit fast execution of movements and thereby slow sprint performance. However, these studies were mainly related to golf putting, not differentiating between execution errors and target errors. More recent reflections on this approach see a fundamental condition in the evaluation of errors rather than in their execution [184], which indicates the importance of corresponding brain states in motor learning. Furthermore, in the sprint context, no studies have currently been published that confirm if these last two approaches also would help to enhance sprint performance.

Approaches that draw on DST to explain performance see the role of the coach shift from a person who gives direct feedback on technique, for example, the coach giving the answer or solution, to a facilitator who creates an interactive learning environment for athletes to solve movement problems. Finally, it is the authors’ opinion that historically, sprint (and sport) coaches have successfully used a spectrum of coaching styles, for example, guided discovery [185], that embody skill acquisition theory, including some of which describe the principles of the original constraints-led approach, yet the labels and terminology now attached to this approach have only more recently been popularised in sport science research.

## Practical Implications

The coaching, teaching and learning concepts discussed in this review have several implications for sprint practitioners. The link between coaching pedagogy/andragogy and the literature to improve sprint performance as evidenced by empirical support to confirm coordination modifications in sprint running is limited. The coordination classification scheme outlined in this review, despite requiring greater expertise for understanding, may provide the only ‘true’ representation that the coordinative patterns observed in sprint performance have been modified, despite a change in sprint outcome, for example, reduced time. Additionally, establishing optimal sprint technique based on group-based analyses, then translating this to individual athletes appears to be an ambitious task for the practitioner. Therefore, this establishes a coaching dilemma of how to provide appropriate coaching instruction at the individual level.

Although we have acknowledged in this review that theoretical concepts such as mechanical degrees of freedom, coordinative structures, constraints and boundary conditions acting on the individual inform sprint coordination and performance, Newell [186] also suggested there are likely informational invariants within a given environment, where consistent patterns or relationships will remain stable for groups or populations of individuals. This assertion by Newell is supported in the research [22, 87, 106], where grouping or clustering athletes with similar movement strategies based on their specific constraints may be a viable method for coaches to prescribe individualised training interventions [187]. Collectively, the authors identified stable intra- and inter-athlete coordinative strategies, where dominant coordination patterns can therefore provide the coach a ‘roadmap’ of where to place their focus to improve sprint performance for each athlete. That is, if there is a dominant or effective coordination strategy for individual sprint performance, greater attention should be placed on creating situations where the athlete can interact within these boundary conditions more frequently. Further, this allows the athlete to train to their ‘coordinative strengths’, thereby reinforcing the individual whole-body kinematic, dynamic and neuromuscular coordinative strategies which elicit positive acceleration and maximal velocity performances. This type of classification framework has previously been explored in sprinting [14, 117, 188] where authors have recommended practitioners use a complementary approach of subjective and objective measures to prescribe sprint training interventions. However, building groups with similar coordination patterns is only an intermediate step on the path towards individuality.

This review endorses the approach that sprint coordinative strategies are individual, and a one-size-fits-all model to achieve ‘optimal’ sprint technique is unachievable [187]. Despite the use of group-based approaches in sport performance coaching, it has been suggested this may have little effect on performance at the inter-athlete level because of unique individual differences, particularly at the elite level [86, 151, 152]. This approach, although grounded in ‘common sense’, may effectively force the athlete to create a coordination pattern that works against the individual intrinsic dynamics and inherent degrees of freedom and movement variability. While a singular universal sprint technique is unachievable because of individual differences in physical capacity and coordination strategies, certain mechanical features consistently emerge in high-performing sprinters. These features can be considered key performance indicators that provide practical reference points when assessing and developing sprint performance. Specifically, effective sprint coordination is commonly characterised by (1) short, aggressive, ground contact times [189] supported by high joint stiffness of the foot–ankle complex (particularly during acceleration) and effective vertical stiffness during maximum velocity phases [190]; (2) a high orientation of horizontal force production [191], accompanied by effective hip extension to project the centre of mass forward [126]; (3) a front-side dominant thigh position at ground contact, contributing to earlier and more effective force application [17, 129]; (4) minimal horizontal braking impulse, reducing deceleration forces [83] and supporting smooth transitions through stance; (5) rapid and coordinated recovery of the swing leg [96, 118], with timing matched to the athlete’s individual action capabilities; (6) stable pelvic projection and orientation, minimising excessive anterior tilt or rotational displacement [159]; and (7) reducing unnecessary tension and improving rhythm and elasticity of movement. These key performance indicators do not prescribe a single technical model but instead reflect common mechanical characteristics around which individual solutions for faster athletes tend to cluster; supporting the views of recent studies [187].

Finally, it appears productive for sprint coaches to align their approach to coaching pedagogy or andragogy and skill acquisition, skill stabilisation or skill refinement with how they view coordination. If coaches acknowledge limitations of cognitive-based perspectives to explain coordinative processes, skill learning approaches embedded in DST may provide alternative and complementary options to not only explain but establish, observe and quantify sprint coordination changes.

## Conclusions

We hope our perspective paper will be viewed as thought provoking to the sprint practitioner, offering a complementary view to traditional approaches of how sprint coordination emerges within applied coaching practice. A recent opinion piece [192] articulated various sentiments of our paper by suggesting there is a need for both behavioural (e.g. environment) *and* cognitive (e.g. central control) perturbations for new coordination patterns to be observed, thereby drawing on a ‘mixed-methods’ approach of DST and information processing skill acquisition perspectives, rather than an ‘all or nothing’ standpoint. Despite a potentially contentious viewpoint, in our opinion, this appears to be the reality amongst practitioners. Additionally, it is suggested perturbations to create new coordination patterns is achieved by the athlete understanding the proposed changes, plus relating this to previous experience (e.g. mental representation) [193]. In addition to these conclusions, it is evident that domain knowledge is critical for practitioners to know both *when* and *how* to use these approaches with their athletes.

This review highlights the overall complexity of understanding coordination patterns in sprinting across the acceleration and maximal velocity phases, plus challenges the concept of one ‘optimal’ sprint technique. For sprint practitioners, the move away from solely reductionist thinking to explain sprint performance towards a contemporary approach to ‘systems’ thinking encourages a more holistic appreciation of how sprint performance emerges over time based on the dynamic interaction of the component parts within the system. Key to this approach is for practitioners to explore a dynamical systems perspective, including concepts such as constraints, boundary conditions, degrees of freedom, optimal technique, the necessity of differences for learning and self-organisation, and their role in sprint coordination and performance. Furthermore, we challenge practitioners to consider drawing on skill acquisition approaches embedded in DST, highlighting the individual nature of how patterns of sprint coordination and performance emerge.

## Footnotes

1 In the context of movement science, boundary conditions refer to natural laws such as gravity, energy conservation, thermodynamics and motion, and define what movement possibilities can occur in a system (the phenomena of interest). These are fundamental principles that govern the behaviour of physical systems. Self-organising coordinative processes occur within the boundary conditions of the human body system. Boundary conditions are different from constraints although often used as synonyms in the literature [35, 36].

2 In this review,“constraints”are used only when organismic (functional or structural/biomechanical) factors *limit or enforce* actions, i.e. they reduce the feasible movement set and thereby shape coordination and control (e.g. by freezing or freeing mechanical degrees of freedom) [51]. This is a more strict application of Newell’s (1986) own definition “Constraints may be viewed as boundaries or features that limit motion of the entity under consideration. In engineering parlance, constraints reduce the number of possible configurations of a system.” When such factors merely specify the context in which the individual acts without forbidding possibilities, we refer to them as **boundary conditions** arising from the individual’s interaction with the environment. Accordingly, organism- and environment-related characteristics are mainly understood as boundary conditions whereas the task category typically defines the constraints for its realisation. Within Sect. 8, there is reference to the ‘constraints led approach’, which is a pedagogical framework using constraints to guide the organism towards an intended solution of a task. [38, 52]

3 To avoid the ambiguity of the term individual, which goes hand in hand with the connotation that on the one hand it denotes an independent living being and on the other hand refers to uniqueness in the forensic sense, the original terminology of Newell, organism (organismic) is used.
